# EBS-seq: enrichment-based method for accurate analysis of 5-hydroxymethylcytosine at single-base resolution

**DOI:** 10.1186/s13148-023-01451-7

**Published:** 2023-03-01

**Authors:** Jaywon Lee, Dongin Lee, Hwang-Phill Kim, Tae-You Kim, Duhee Bang

**Affiliations:** 1 Department of Chemistry, Yonsei University, Seoul, Korea; 2 IMBdx, Seoul, Korea; 3 Department of Internal Medicine, Seoul National University Hospital, Seoul, Korea

**Keywords:** 5-hydroxymethyl cytosine, Colorectal cancer, Detection, Enrichment

## Abstract

**Background:**

A growing body of research has emphasized 5-hydroxymethylcytosine (5hmC) as an important epigenetic mark. High-resolution methods to detect 5hmC require high sequencing depth and are therefore expensive. Many studies have used enrichment-based methods to detect 5hmC; however, conventional enrichment-based methods have limited resolution. To overcome these limitations, we developed EBS-seq, a cost-efficient method for 5hmC detection with single-base resolution that combines the advantages of high-resolution methods and enrichment-based methods.

**Results:**

EBS-seq uses selective labeling of 5hmC, deamination of cytosine and 5-methylcytosine, pull-down of labeled 5hmC, and C-to-T conversion during DNA amplification. Using this method, we profiled 5hmC in HEK293T cells and two colorectal cancer samples. Compared with conventional enrichment-based 5hmC detection, EBS-seq improved 5hmC signals by localizing them at single-base resolution. Furthermore, EBS-seq was able to determine 5hmC levels in CpG-dense regions where distortion of signals can occur, such as CpG islands and CpG shores. Comparing EBS-seq and conventional high-resolution 5hmC detection by ACE-seq, we showed that EBS-seq is more effective at finding 5hmC sites. Using EBS-seq, we found strong associations between gene expression and gene-body 5hmC content in both HEK293T cells and colorectal cancer samples.

**Conclusions:**

EBS-seq is a reliable and cost-efficient method for 5hmC detection because it simultaneously enriches 5hmC-containing DNA fragments and localizes 5hmC signals at single-base resolution. This method is a promising choice for 5hmC detection in challenging clinical samples with low 5hmC levels, such as cancer tissues.

**Supplementary Information:**

The online version contains supplementary material available at 10.1186/s13148-023-01451-7.

## Background

An oxidative form of cytosine called 5-hydroxymethylcytosine (5hmC) is found in various mammalian cells such as human brain cells [1] and mouse embryonic stem cells [2]. In human cells, 5hmC is produced by oxidation of 5-methylcytosine (5mC) by ten-eleven translocation (TET) enzymes during active DNA demethylation [3]. The 5hmC is further oxidized by TET enzymes to form 5-formylcytosine (5fC) and 5-carboxylcytosine (5caC) and turned into unmodified cytosine by a base excision-repair pathway [4]. Recent studies suggested that 5hmC may have important roles in biological processes such as cell differentiation and cancer development [5, 6]. In addition, strong enrichment of 5hmC in gene bodies and regulatory regions suggests 5hmC plays a role in transcription activity [7]. Two recent studies profiled the landscape of 5hmC in diverse tissues to understand its regulatory functions and found strong correlations between gene-body 5hmC levels and gene expression [8, 9].

Although 5hmC content varies greatly among tissue types [10], 5hmC is strongly depleted in cancer tissues and human cell lines [11, 12]. Therefore, 5hmC has been investigated as a potential marker for early cancer detection in diverse clinical samples such as blood [13, 14]. Profiling of 5hmC using high-resolution sequencing methods such as oxidative bisulfite sequencing (oxBS-Seq) [15], TET-assisted bisulfite sequencing (TAB-Seq) [16], chemical-assisted pyridine borane sequencing (CAPS) [17], and APOBEC-coupled epigenetic sequencing (ACE-seq) [18] is too expensive for clinical application because of the large amounts of sequencing data that are required. On the other hand, enrichment-based sequencing methods such as 5hmC antibody-based hydroxymethylated DNA immunoprecipitation (hMEDIP-seq) [19] and selective 5hmC chemical labeling (hmC-Seal) [20] lack the ability to localize 5hmC at single-base resolution. To address these limitations, there are techniques that simultaneously enrich and identify 5hmC at high resolution. Jump-seq, for example, can enrich and indirectly detect 5hmC at nearly single-base resolution [21]. Another technique, chemical-assisted C-to-T conversion of 5hmC sequencing (hmC-CATCH), utilizes chemical reactions to selectively enrich and identify 5hmC at single-base resolution [22]. Recently, a new technique that combines hmC-SEAL and ACE-seq was published [23]. This technique employs a similar workflow to our method, but there are some differences in details such as the adapter ligation process. Furthermore, the research only evaluated its technique using genomic DNA of mouse embryonic stem cells, and it remained uncertain if the technique can be applied to clinical samples.

A gold standard for detecting 5hmC has not yet been determined, and there is a need for more advanced techniques. Here, we propose an enrichment-based, single-base resolution 5hmC sequencing (EBS-seq) that utilizes T4 Phage β-glucosyltransferase to tag 5hmC and APOBEC deaminase to discriminate modified cytosines from unmodified cytosines. EBS-seq can simultaneously enrich 5hmC-containing DNA fragments and localize 5hmCs at single-base resolution. To show the advantage of EBS-seq for samples with low levels of 5hmC, we used EBS-seq to profile 5hmC in HEK293T cells, which generally show low levels of 5hmC [12]. In addition, because gene-body 5hmC content is correlated with gene expression in many types of mammalian tissues [9, 24], we measured gene expression in HEK293T cells by RNA-seq and compared it to gene-body 5hmC content detected by EBS-seq. Finally, we performed EBS-seq with two human colorectal cancer (CRC) samples and found strong associations between gene expression and gene-body 5hmC levels.

## Results

### The EBS-seq design

**Fig. 1:**
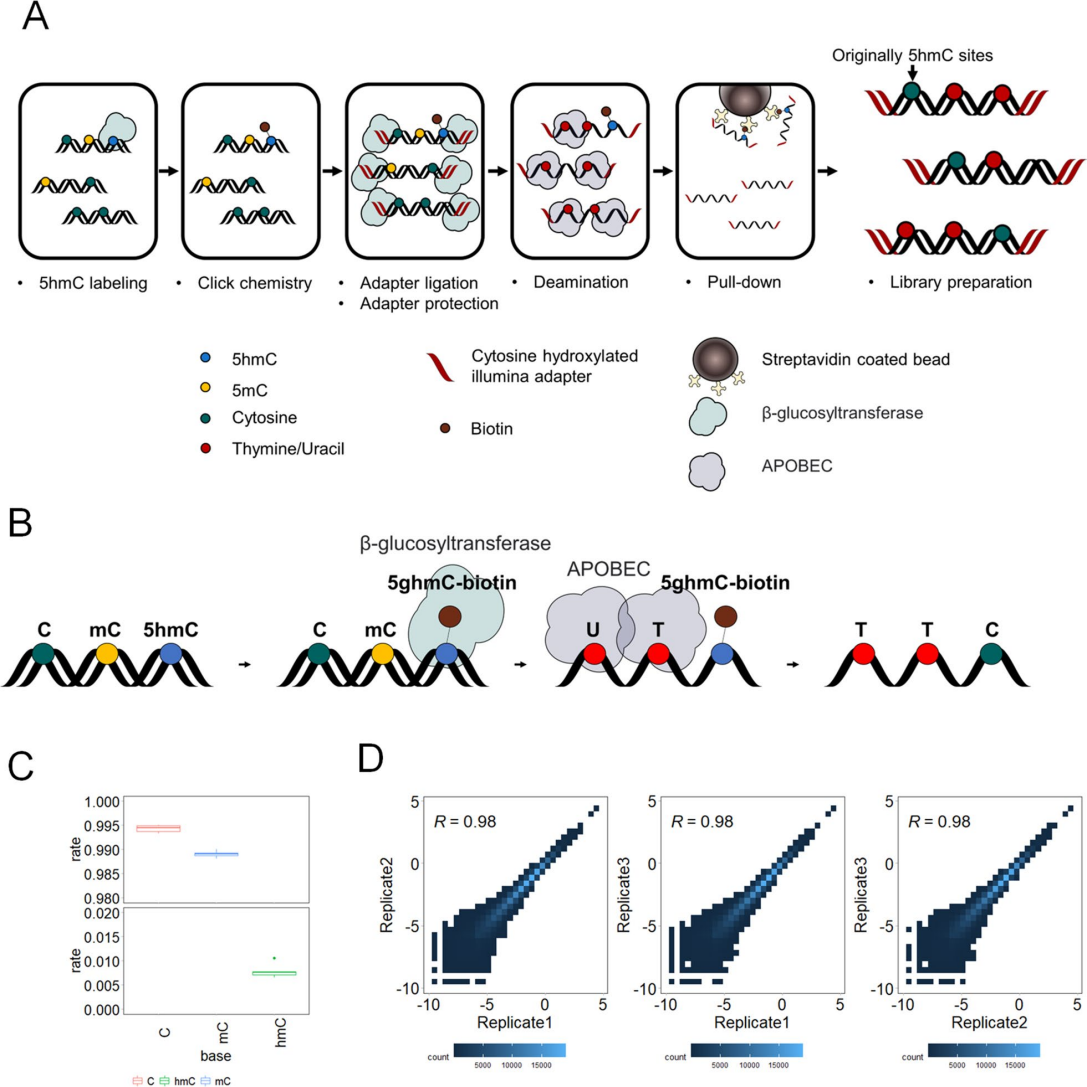
Enrichment-based single-base resolution 5hmC sequencing (EBS-seq) method. **A** Schematic of the EBS-seq method. **B** Illustration of selective 5hmC labeling and deamination of unmodified cytosine and 5mC. **C** Deamination efficiency of cytosine, 5-methylcytosine, and 5-hydroxymethylcytosine in spike-in DNA. **D** Heatmap showing correlations between replicates. Heatmap was created using RPKM-normalized read counts within sliding 10 kb windows. Correlation coefficients were calculated using Pearson correlation

EBS-seq is an organized combination of library preparation techniques and 5hmC detection methods (Fig. 1A). EBS-seq uses a two-step 5hmC labeling method to selectively label and block 5hmCs in DNA fragments [20]. 5hmC undergoes glycosylation with T4 Phage β-glucosyltransferase, and then is biotinylated using click chemistry. Specially modified Illumina-compatible adapters (Additional file 6: Table S1), in which every cytosine is replaced with 5hmC, are ligated to both ends of DNA fragments. The modified cytosines in the adapters are protected from the deamination reaction that follows (see the Methods). During the deamination reaction, only 5mC and unmodified cytosine, but not 5hmC, 5fmC or 5caC, are deaminated by APOBEC (Fig. 1B) [18, 25]. Although the presence of 5fmC and 5caC modifications is very low in genomic DNA, 5fC and 5caC will not be deaminated by APOBEC and could contribute to unmodified Cs after EBS-seq. DNA fragments containing 5hmC are then selectively enriched using streptavidin-coated beads. Libraries are generated from the enriched fragments by PCR amplification with a uracil-tolerant polymerase. Unmodified cytosines and 5mCs that have been deaminated are converted to thymines in this step. The libraries are then subjected to Illumina high-throughput sequencing.

We tested the deamination reaction using a mixture of CpG-methylated pUC19 plasmid and an unmodified lambda plasmid. After filtering out the non-converted strand, we found that commercial APOBEC could readily convert 5mCs (97.4%) and unmodified cytosines (99.6%) (Additional file 7: Table S2) in CpGs. Cytosines in other contexts also showed very high conversion efficiency (over 99.5% in all replicates).

### Validation of the EBS-seq

To validate the deamination efficiency and enrichment of 5hmC, two spike-in DNA were utilized (see method, Additional file 6: Table S1). The two spike-in DNA were highly similar, with one containing 5hmC and the other lacking this modification. We performed triplicates of EBS-seq on a sample of CRC that had been spiked with equal amounts of annealed DNA spike-ins. The EBS-seq process successfully enriched the spike-in DNA strand that contained 5hmC, as over 97% of the spike DNA present in the sequencing data were found to be this strand in all the samples (Additional file 8: Table S3). We calculated the deamination efficiency of EBS-seq by analyzing the conversion rates of unmodified cytosines, 5-methylcytosines, and 5-hydroxymethylcytosines present in 5hmC-containing spike-in DNA. The overall C-to-T conversion rate was high for unmodified cytosines (99.4%) and 5-methylcytosines (98.9%), but low for 5-hydroxymethylcytosines (0.8%) (Fig. 1C). Because the impact of the bulky modification of 5hmC on the deamination of adjacent cytosines was previously unknown, we further analyzed the deamination efficiency at each cytosine position in the spike-in DNA. We found a slight decrease in conversion efficiency at adjacent unmodified cytosines (99%), but this difference was small (see Additional file 1: Fig. S1A, B). These results suggest that EBS-seq can effectively enrich and detect 5hmC at single-base resolution.

Unexpectedly, we observed large fraction of read pairs that did not contain any unconverted cytosines which may be remnant DNA fragments that escaped removal even after thorough selective pull-down for 5hmC (Additional file 9: Table S4). After further analysis to identify possible explanations, we found that the reads were not completely random. Instead, they were found to have peaks of enrichment in areas where 5hmC is commonly found (Additional file 1: Fig. S1C) [8, 9]. This suggests that at least some of these molecules may have a connection to 5hmC. One potential explanation for this observation is that these read pairs are from DNA fragments containing 5hmC at the center of the strand, which are not covered by the Illumina 150PE platform. The average insert size of read pairs without 5hmC were longer than that of read pairs with 5hmC which might support this explanation (Additional file 1: Fig. S1D). However, we could not rule out the possibility that these are the results of remnant DNA fragments that escaped removal during the wash step. We removed these read pairs to ensure that all reads in the subsequent analysis were genuine 5hmC pull-down signals. After removing read pairs without 5hmC, the EBS-seq results were highly reproducible among the replicates. We calculated the Pearson correlation coefficients of normalized 5hmC signals in 10 kb sliding windows across the genome between the replicates and found that the coefficients between all replicates were high, with an average of 0.98 (Fig. 1D). This suggests that EBS-seq is highly reproducible.

### Metagene 5hmC analysis of HEK293T cells

**Fig. 2:**
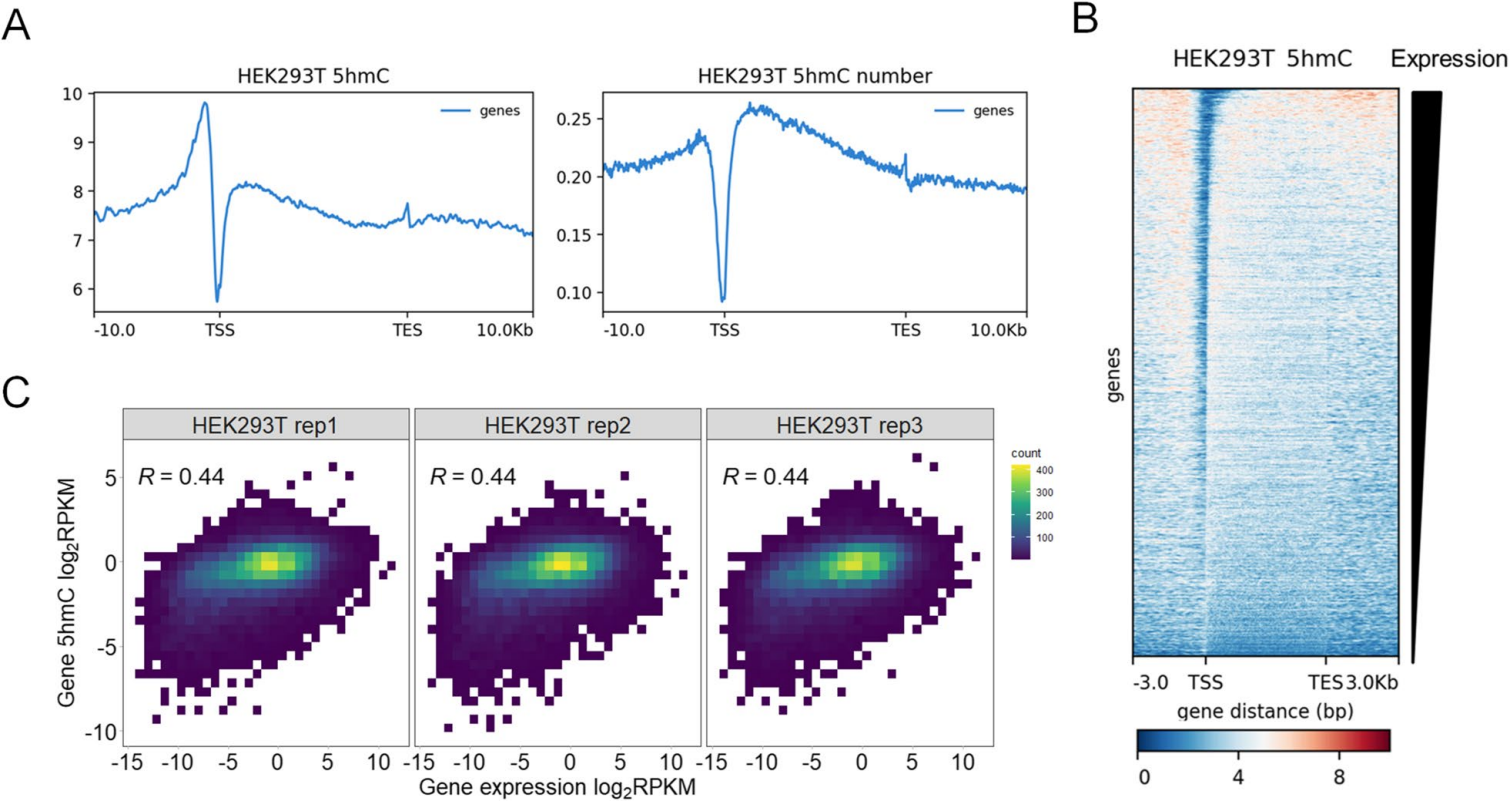
Metagene profiling of 5hmC shows enrichment of 5hmC in gene bodies. Gene-body 5hmC content is associated with gene expression in HEK293T cells. **A** Metagene analysis using normalized coverage (left) and average number of 5hmCs at CpG sites (right) in HEK293T cells. *Y*-axis shows normalized reads coverage (left) and number of detected 5hmCs (right). **B** Heatmap showing the 5hmC distribution on individual genes in decreasing order of expression. The colors represent normalized reads coverage. **C** Heatmaps showing correlations with correlation coefficients between gene expression and gene-body 5hmC content in HEK293T cells. Heatmap was created using RPKM-normalized read counts within gene bodies. Normalization was performed using the RPKM method. Correlation coefficients were calculated by Pearson correlation

To evaluate the performance of EBS-seq, we performed EBS-seq with cultured HEK293T cells, which show low levels of 5hmC. Triplicate EBS-seq libraries were generated. After removing reads that did not contain any unconverted cytosines, we could visualize 5hmCs more clearly (Additional file 2: Fig. S2A, Fig. 2A) [26]. Using EBS-seq, we found that 5hmC in HEK293T cells was enriched in gene bodies and promoter regions and depleted in intergenic regions (Additional file 2: Fig. S2B), which is consistent with previous studies that utilized enrichment strategies to identify 5hmC-enriched regions [8, 9]. Metagene analysis of normalized reads coverage showed that reads containing 5hmCs were highly enriched in regions ~ 3 kb upstream from transcription start sites, which are CpG-dense promoter regions rather than gene bodies (Fig. 2A, left). However, in 5hmC enrichment, the regional reads coverage can be affected by the presence of CpG sites (see Additional file 3: Fig. S3). Accordingly, previous 5hmC studies that used conventional enrichment-based strategies showed 5hmC enrichment in promoter regions, CpG shores, and CpG islands where CpG sites are abundant [27–30]. Although it is not possible to quantitatively estimate the ratio of 5hmC to unmodified cytosine at a single CpG site with EBS-seq, we plotted the numbers of detected 5hmCs at each CpG site around genes (Fig. 2A, right; see the Methods). Compared with the reads coverage, which can be affected by CpG density (Fig. 2A, left), the number of detected 5hmCs showed smaller 5hmC signals around the regions upstream of transcription start sites, suggesting that the reads coverage of these regions is strongly affected by the CpG density. We also found that 5hmCs were overestimated at the boundaries of CpG islands in the reads coverage (Additional file 4: Fig. S4A).

### 5hmC content correlates with gene expression in HEK293T cells

Recent studies found strong correlations between gene expression and gene-body 5hmC levels in human and mouse embryonic stem cells [8, 9, 24]. We subjected genomic DNA and mRNA of HEK293T cells to EBS-seq and RNA-seq to determine whether this correlation is present in a human cell line with low 5hmC content. We counted the 5hmC reads that mapped to gene-body regions and normalized the read counts using the RPKM method (see the Methods). We observed a strong correlation (*R* = 0.43, Pearson correlation coefficient) between the gene expression and gene-body 5hmC content (Fig. 2B, C). Before reads without 5hmC signals were removed, the correlation coefficients between gene-body 5hmC content and gene expression were low and inconsistent at 0.26, 0.31, and 0.23 in the three replicates, respectively, which suggests that the filtering step is important for profiling 5hmC levels (Additional file 4: Fig. S4B). When we grouped genes according to their expression levels, we observed that most of the silenced genes had very low gene-body 5hmC levels, whereas highly expressed genes had higher gene-body 5hmC levels (Additional file 4: Fig. S4C). This trend was less obvious before the reads with no 5hmCs were removed (Additional file 4: Fig. S4D). These observations suggest that the ability of EBS-seq to detect 5hmCs at single-base resolution (and remove noise reads) is advantageous for finding associations between gene expression and gene-body 5hmC content.

Cultured human cells have substantially lower 5hmC levels than human tissue samples, presumably because of passive loss of 5hmC in proliferating cells or of a lack of ascorbic acid, which is essential for TET function, in cell culture media [31–33]. We demonstrated that EBS-seq can effectively profile 5hmC in challenging samples with low level of 5hmC, by successfully profiling 5hmC in the cultured human cell line HEK293T.

### EBS-seq is more cost-efficient than conventional high-resolution 5hmC profiling

Depletion of 5hmC in tumors compared with corresponding normal tissues is observed in various cancer types [11, 34, 35], including CRC [12, 36, 37]. Conventional high-resolution 5hmC detection methods such as TAPS or ACE-seq are often inefficient for clinical samples. One critical reason for this is that the sequencing cost for reliable 5hmC detection increases as the level of 5hmC decreases. To directly compare EBS-seq with conventional high-resolution 5hmC sequencing, we performed ACE-seq with one of our CRC samples. We first compared the average 5hmC levels determined by ACE-seq to the EBS-seq RPKM values and found that genome-wide correlations between the two methods were observable (Additional file 5: Fig. S5A). However, in enrichment-based 5hmC detection, RPKM values can be affected by CpG density. For that reason, we normalized the EBS-seq 5hmC signals by dividing the total number of 5hmCs per million 5hmCs detected by the total number of CpG sites in the region. After this normalization, the calculated correlation coefficients between ACE-seq and EBS-seq were higher, suggesting that the normalization improved the representation of the CpG density-independent 5hmC signal (Additional file 5: Fig. S5B).

**Fig. 3:**
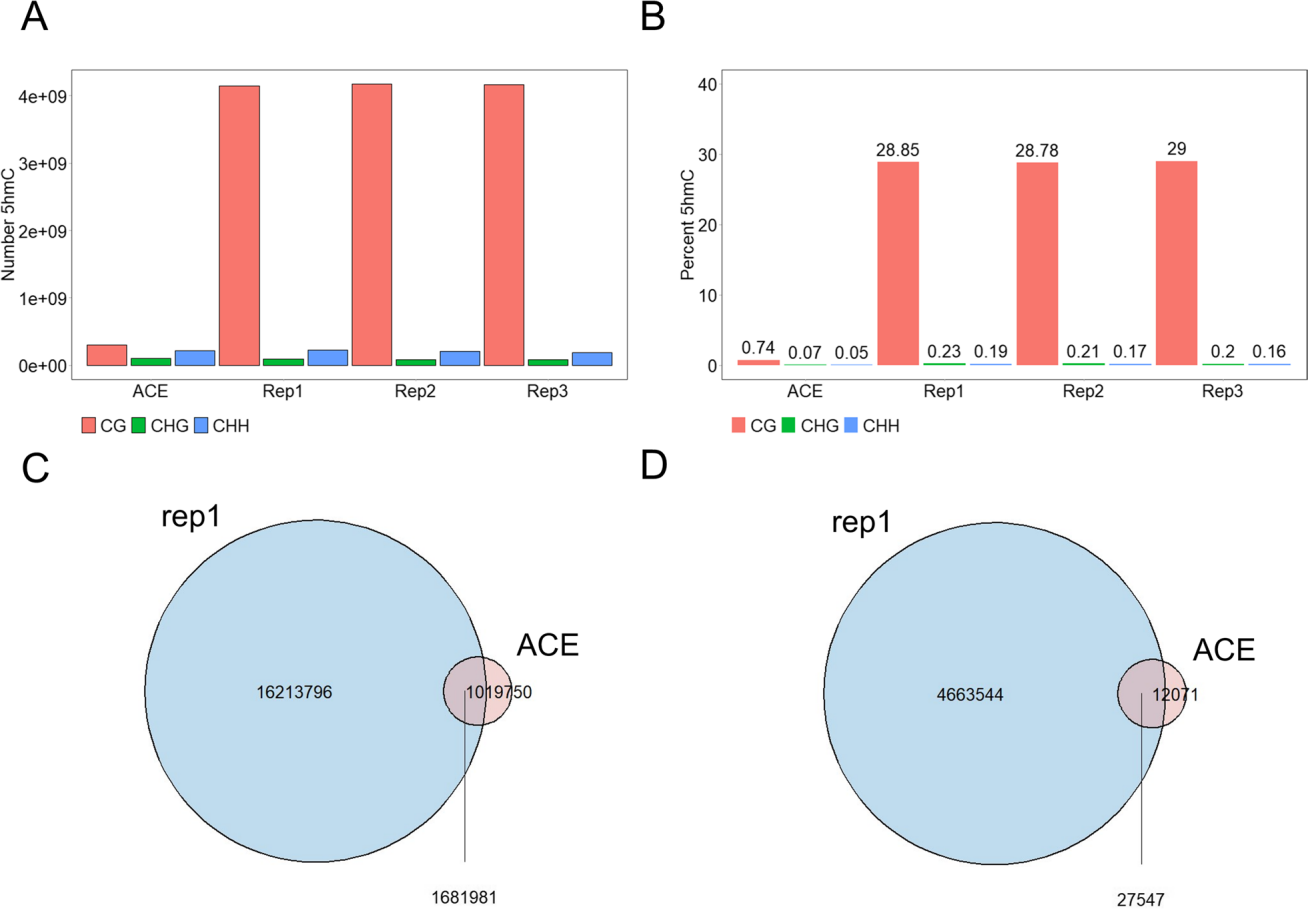
EBS-seq is more effective for detecting 5hmCs than ACE-seq, a conventional method for high-resolution 5hmC detection. **A** Bar chart showing the number of 5hmC signals detected by ACE-seq and three replicates of EBS-seq in three cytosine contexts. **B** Bar chart showing the proportion of 5hmC signals detected by ACE-seq and three replicates of EBS-seq in each cytosine context across the whole genome. **C**, **D** EBS-seq was able to identify a larger number of CpG sites when compared to ACE-seq. EBS-seq detected more (**C**) CpG sites overall, and (**D**) high-confidence CpG sites that have more than three 5hmC signals

Despite the similar number of reads (260 M raw read pairs for ACE-seq, 250 M, 240 M, and 236 M raw read pairs for each EBS-seq replicate, respectively), the raw 5hmC signals in ACE-seq, which are cytosines read as ‘C’ in CpG positions, were much smaller than those in EBS-seq (Fig. 3A). In fact, the percentage of unconverted cytosines that read as ‘C’ in CpG positions was only 0.744% in the ACE-seq library, indicating a very low level of 5hmCs in the CRC sample (Fig. 3B). By contrast, the percentage of 5hmC signals in CpG sites detected by EBS-seq was higher than 28% in every replicate. However, it is important to be aware that EBS-seq is an enrichment-based method and does not provide an accurate quantification of the absolute level of 5hmC.

Also, about 62% of CpG sites with one or more 5hmC signals detected by ACE-seq were also discovered by EBS-seq, whereas only about 9% of CpG sites detected by EBS-seq were detected by ACE-seq (Fig. 3C). To compare only high-confidence 5hmC sites, we compared only CpG sites with at least three 5hmC signals. About 70% of the high-confidence 5hmC sites detected by ACE-seq were detected by EBS-seq, whereas less than 1% of high-confidence 5hmC sites detected by EBS-seq were detected by ACE-seq (Fig. 3D). The comparisons between EBS-seq replicates are shown in Additional file 5: Fig. S5C, D.

### Gene-body 5hmC content in CRC samples correlates with gene expression

**Fig. 4:**
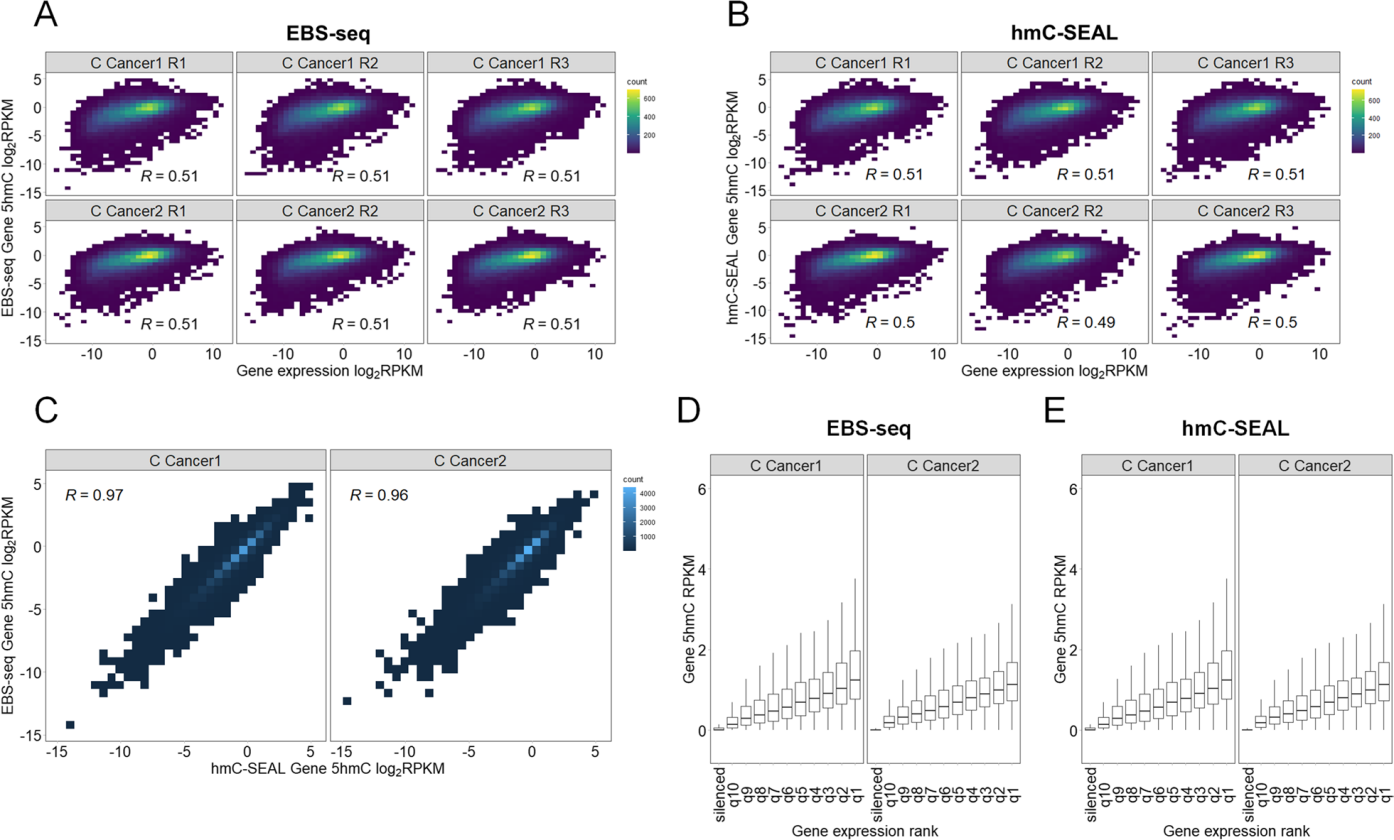
Gene-body 5hmC is associated with gene expression in CRC. Heatmaps illustrating the relationship between gene expression and gene-body 5hmC content were created from (**A**) EBS-seq data and (**B**) hmC-SEAL data. Heatmap was created using RPKM-normalized read counts within gene bodies. Correlation coefficients were calculated using Pearson correlation. **C** Heatmap showing correlations between EBS-seq and hmC-SEAL. Heatmap was created using RPKM-normalized read counts within gene bodies. Correlation coefficients were calculated using Pearson correlation. **D**, **E** Box plots showing gene-body 5hmC content in each CRC sample according to gene expression by utilizing (**D**) EBS-seq data and (**E**) hmC-SEAL data

To show the utility of EBS-seq for clinical samples, we attempted to find associations between gene-body 5hmC content and gene expression in two CRC samples. For this, we calculated the Pearson correlation coefficient between the 5hmC counts determined by EBS-seq and the RPKM-normalized RNA-seq counts from a previous human CRC transcriptome study (see the Methods) [38]. The correlation coefficient between gene expression and gene-body 5hmC content was high (Fig. 4A).

We applied hmC-SEAL, an enrichment-based sequencing method with limited resolution, on the same sample to compare it with EBS-seq. The correlation coefficient calculated using hmC-SEAL was very similar to EBS-seq; however, it was slightly lower in one of the replicates (C Cancer 2 R2, Fig. 4B). Despite this, the correlation between gene-body 5hmC detected by two method was still high (*R* = 0.96, Pearson correlation coefficient) (Fig. 4C). Furthermore, we observed the same pattern of 5hmC depletion in the gene bodies of silenced genes in both CRC samples using both methods, which is consistent with previous experiments using HEK293T cells (Fig. 4D, E).

These data demonstrate that EBS-seq is a reliable technique that preserves the information of enrichment-based detection methods such as hmC-SEAL, but also has the added benefit of being able to sequence 5hmC at single-base resolution.

## Discussion

EBS-seq is an enrichment-based method for 5hmC detection with single-base resolution that efficiently detects 5hmC in samples with low 5hmC content, such as cultured human cell lines and clinical cancer samples. All of the reagents used for EBS-seq are commercially available (see the Methods for sources), making EBS-seq easy to reproduce. One of the key challenges for 5hmC detection with single-base resolution is the sequencing cost. Levels of 5hmC are typically much lower than levels of 5mC, making 5hmC costly to detect with conventional high-resolution sequencing. A comparison of EBS-seq with ACE-seq showed that EBS-seq can detect 5hmC more efficiently than ACE-seq by enriching DNA fragments containing 5hmC.

EBS-seq can localize 5hmC at single-base resolution, making it possible to filter out irrelevant reads in silico. Unexpectedly, we observed that a large fraction of reads (40–88%) in enriched libraries do not have any 5hmC signals (Additional file 9: Table S4). The source of read pairs without 5hmC were not entirely clear, but this was especially notable in samples from HEK293T cells, which required large amounts of DNA fragments to generate a library because of the low 5hmC levels in the genomic DNA. The use of large amounts of DNA may cause non-specific binding of DNA fragments to streptavidin beads or the surface of tubes used to hold the samples. Clarifying the origin of unwanted DNA in addition to optimizing the washing procedure is necessary to decrease the number of undesired reads in the library and increase cost efficiency. Nevertheless, EBS-seq avoids this problem by filtering out such unwanted DNA molecules, giving it an advantage over other enrichment-based 5hmC detection methods.

During the time that our research was being conducted, a new technique that combines hmC-SEAL and ACE-seq was published [23]. The approach was similar to EBS-seq, but there were also notable distinctions. In the study published, the adapters were ligated to the pull-down DNA, which is in trace amount, after the pull-down step. According to the study authors, this caused a lower mapping ratio (61%) which is much lower than EBS-seq (88%) (Additional file 10: Table S5). Furthermore, the study only tested their technique on mouse embryonic stem cells, whereas we tested EBS-seq on CRC to demonstrate its utility on clinical samples.

Many recent studies have used enrichment-based strategies to profile 5hmC, but the quality of the 5hmC signal depends on technical skills to wash the pull-down beads and on the total content of 5hmC in the samples. Profiling of 5hmC with EBS-seq provides more stable and reliable detection than conventional enrichment-based methods and can thus improve our understanding of the role of 5hmC in diverse types of clinical samples.

## Conclusion

Studies have emphasized 5hmC as an important epigenetic mark in clinical samples; however, a gold standard for 5hmC detection has not been established. We present EBS-seq as a competitive 5hmC sequencing method. We show that EBS-seq can efficiently and confidently detect 5hmCs in HEK293T cells and CRC samples. We hope that EBS-seq can contribute to future research on 5hmC.

## Materials and methods

### Cell culture and extraction of genomic DNA and total RNA from cultured cells and tumor samples

The HEK293T cell line was obtained from the Korean Cell Line Bank. HEK293T cells were cultured in Dulbecco’s modified Eagle’s medium (Gibco) with 10% fetal bovine serum (Gibco) and 1% penicillin/streptomycin (Thermo Fisher Scientific) at 37 °C with 5% CO_2_. Genomic DNA was extracted using DNeasy Blood & Tissue Kits (QIAGEN) according to the manufacturer's instructions. Total RNA was extracted using RNeasy Mini Kits (QIAGEN) according to the manufacturer's instructions. Genomic DNA was extracted from two fresh frozen CRC tissues using DNeasy Blood & Tissue Kits (QIAGEN) according to the manufacturer's instructions.

### RNA-seq library preparation

The quality of total RNA extracted from HEK293T cells was confirmed using an Agilent 4200 TapeStation System (Agilent). Removal of rRNA and isolation of mRNA were performed using a NEBNext^®^ Poly(A) mRNA Magnetic Isolation Module (New England Biolabs; Catalog #E7490S) according to the manufacturer's protocol. The RNA library was constructed using NEBNext^®^ Ultra™ II RNA Library Prep Kit for Illumina^®^ (New England Biolabs; Catalog #E7770S) according to the manufacturer's protocol.

### EBS-seq library preparation

Genomic DNA was sheared into fragments with an average size of 200 bp using an M220 Focused-Ultrasonicator (Covaris) according to the manufacturer’s instructions. The fragmented DNA was measured using an Agilent 4200 TapeStation System (Agilent). Four micrograms of fragmented DNA from HEK293T cells or 50 ng fragmented DNA from tumor tissues were treated with T4 Phage β-glucosyltransferase (New England Biolabs; Catalog #M0357S) and 60 μM UDP-6-azide-glucose (Jena Bioscience) in 1X NEBuffer™ 4 (New England Biolabs; Catalog #B7004S) at 37 °C for 1 h. Then, 2 μL DBCO-PEG4-Biotin Conjugate (Jena Bioscience, 20 mM stock in DMSO) was added to the reaction mixture and incubated at 37 °C for 2 h. The modified DNA was then purified using AMPure XP beads. Next, modified Illumina-compatible adapters (Additional file 6: Table S1) (BIONEER) were ligated to the DNA fragments using a NEBNext^®^ Ultra™ II DNA Library Prep Kit for Illumina^®^ (New England Biolabs; Catalog #E7645S) according to the manufacturer’s protocol. The adapter-ligated DNA was then treated with T4 Phage β-glucosyltransferase (New England Biolabs; Catalog #M0357S) and UDP-Glucose in 1X NEBuffer supplied with the enzyme in a total reaction volume of 16 μL at 37 °C for 1 h. Next, 4 μL formamide was added, and the mixture was incubated at 80 °C for 10 min and then immediately put on ice. The denatured DNA was then subjected to enzymatic deamination using a NEBNext^®^ Enzymatic Methyl-seq Kit (New England Biolabs; Catalog #E7120S). The converted DNA was then incubated with 5 μl Dynabeads™ MyOne™ Streptavidin C1 (Invitrogen) in 1X binding and washing buffer (5 mM Tris–HCl (pH 7.5)) 500 μM EDTA, 1 M NaCl) at room temperature for 15 min. The beads were then washed with 1X binding and washing buffer five times. HEK293T samples underwent another round of enzymatic deamination for complete conversion of cytosines and 5-methyl cytosines. Enriched DNA was amplified with 12 cycles of PCR using NEBNext Q5U Master Mix supplied in the NEBNext^®^ Enzymatic Methyl-seq Kit. Libraries were sequenced on an Illumina Novaseq6000 platform to generate paired-end data.

### ACE-seq library preparation from CRC samples

We simplified the ACE-seq protocol using the modified Illumina-compatible adapters described above. First, the modified adapters were ligated to 50 ng sheared DNA using the NEBNext^®^ Ultra™ II DNA Library Prep Kit for Illumina^®^ (New England Biolabs; Catalog #E7645S) according to the manufacturer’s protocol. The adapter-ligated DNA was then treated with T4 Phage β-glucosyltransferase (New England Biolabs; Catalog #M0357S) and UDP-Glucose in 1X NEBuffer supplied with the enzyme in a total reaction volume of 16 μL at 37 °C for 1 h. Next, 4μL formamide was added, and the mixture was incubated at 80 °C for 10 min and then immediately put on ice. The denatured DNA was then subjected to enzymatic deamination using a NEBNext^®^ Enzymatic Methyl-seq Kit (New England Biolabs; Catalog #E7120S). The DNA was then amplified with six cycles of PCR using NEBNext Q5U Master Mix supplied in the NEBNext^®^ Enzymatic Methyl-seq Kit. Libraries were sequenced on the Illumina Novaseq6000 platform to generate paired-end data.

### hmC-SEAL library preparation from CRC samples

50 ng fragmented DNA from tumor tissues were treated with T4 Phage β-glucosyltransferase (New England Biolabs; Catalog #M0357S) and UDP-6-azide-Glucose (Jena Bioscience) in 1X NEBuffer supplied with the enzyme (New England Biolabs; Catalog #B7004S) at 37 °C for 1 h. Then, 2 μL DBCO-PEG4-Biotin Conjugate (Jena Bioscience, 20 mM stock in DMSO) was added to the reaction mixture and incubated at 37 °C for 2 h. The modified DNA was then purified using AMPure XP beads. Next, TruSeq adapters were ligated to the DNA fragments using a NEBNext^®^ Ultra™ II DNA Library Prep Kit for Illumina^®^ (New England Biolabs; Catalog #E7645S) according to the manufacturer’s protocol. The DNA fragments with 5hmC were enriched as described above. Enriched DNA was amplified with 12 cycles of PCR using NEBNext Q5U Master Mix supplied in the NEBNext^®^ Enzymatic Methyl-seq Kit. Libraries were sequenced on an Illumina Novaseq6000 platform to generate paired-end data.

### RNA-seq data processing

Adapter sequences and low-quality reads were removed using fastp [39]. The trimmed reads were then aligned using hisat2 [40] to the hg19 human genome [41]. Uniquely mapped reads were used for downstream analysis. Reads were counted on exons using FeatureCounts [42].

### EBS-seq data processing and analysis

Adapter sequences and low-quality reads were removed using fastp [39]. The trimmed reads were then aligned to the hg19 human genome using Bismark aligner [43]. Reads with more than three unconverted cytosines in the context of non-CG motifs were discarded to eliminate false positives due to incomplete deamination of cytosine. PCR duplicates were discarded using GATK [44], and the remaining reads were used for downstream analysis.

Coverage bigWig files were generated for each sample using deeptools bamCoverage [45] and visualized using igv [26]. MACS2 [46] was used to call 5hmC-enriched regions (peaks) in processed bam files. The peaks were annotated according to their genomic features using HOMER annotatepeaks.pl [47] with hg19 as the reference. Reads were counted in 10 kb bins using bedtools [48], and the counts were used to calculate Pearson correlation coefficients between replicates. Reads were counted specifically in exons using FeatureCounts [42] for comparison with RNA-seq data. Genes without any mapped read counts in either EBS-seq or RNA-seq were excluded from the correlation analysis.

### EBS-seq metagene analysis

We used MethylDackel extract [49] to extract 5hmC information into bedgraph for each CpG site with the—cytosine_report option. Average coverage and average number of 5hmCs at CpG sites were calculated around gene bodies and CpG islands using deeptools computeMatrix and plotProfile [45].

### ACE-seq data analysis

Adapter sequences and low-quality reads were removed using fastp [39]. The trimmed reads were then aligned to the hg19 human genome using Bismark aligner [43]. Reads with more than three unconverted cytosines in the context of non-CG motifs were discarded to eliminate false positives due to incomplete deamination of cytosine. PCR duplicates were discarded using GATK [44], and the remaining reads were used for downstream analysis. We used MethylDackel extract [49] to extract 5hmC information for each CpG site with the—cytosine report option.

### hmC-SEAL data analysis

Adapter sequences and low-quality reads were removed using fastp [39]. The trimmed reads were then aligned to the hg19 human genome using Bismark aligner [43]. PCR duplicates were discarded using GATK [44], and the remaining reads were used for downstream analysis. Reads were counted specifically in exons using FeatureCounts [42] for comparison with RNA-seq data. Genes without any mapped read counts in either hmC-SEAL or RNA-seq were excluded from the correlation analysis.

### Spike-in control validation analysis

Two double-stranded spike-in DNA were created by annealing spike-in DNA oligos (Integrated DNA Technologies) (Additional file 6: Table S1). 1fmol of each double-stranded spike-in DNA were spiked into 50 ng of fragmented CRC genomic DNA for EBS-seq. After adapter trimming, spike-in DNA read pairs were extracted and used for the enrichment and deamination efficiency analysis.

### Comparison between gene-body 5hmC and transcriptome in CRC

The raw RNA-seq data of the CRC transcriptome study on human colorectal cancer was obtained from an external source and processed as described above [38]. Uniquely mapped reads were used for downstream analysis. Reads were counted on exons using FeatureCounts [42]. For hmC-SEAL and EBS-seq data, reads were counted specifically in exons using FeatureCounts for comparison with RNA-seq data [42]. Genes without any mapped read counts in either enrichment methods (EBS-seq and hmC-SEAL) or RNA-seq were excluded from the correlation analysis. Read counts were normalized using the RPKM method.

## Supplementary Information

**Additional file 1**: **Fig. S1**. (A) Deamination efficiency of each cytosine position in spike-in DNA. (B) Deamination efficiency of cytosines adjacent to modified 5hmC. (C) Genomic distribution of 5hmC peaks of read pairs without 5hmC. (D) Average insert size of read pairs with and without 5hmC.

**Additional file 2**: **Fig. S2**. (A) Integrative Genome Browser visualization of improved 5hmC signals of HEK293T cells in the Chr2:114,883,671–131,429,913 region. 5hmC-depleted regions are marked with yellow. Gray plots represent signals before filtering; blue plots represent signals after filtering. (B) Genomic distribution of 5hmC peaks showing enrichment in promoters and gene bodies in genomic DNA of HEK293T.

**Additional file 3**: **Fig. S3**. Illustration of possible distortion caused by different regional CpG densities when using sequencing coverage to evaluate 5hmC levels.

**Additional file 4**: **Fig. S4**. (A) 5hmC profiles around CpG islands using normalized coverage (left) and average 5hmC numbers (right) in HEK293T cells. (B) Heatmaps showing lower correlations between gene expression and gene-body 5hmC content before noise filtering. Heatmap was created using RPKM-normalized read counts within gene bodies. Normalization was performed using the RPKM method. We observed stronger depletion of 5hmC in silenced genes (red arrow) (C) after filtering out reads without 5hmC signals than (D) before filtering.

**Additional file 5**: **Fig. S5**. (A) Scatter plots showing correlations between EBS-seq and ACE-seq results for CRC tissue. Each dot represents an RPKM-normalized read count (EBS-seq) or a 5hmC proportion (ACE-seq) within sliding 100kb windows. Correlation coefficients were calculated by Pearson correlation. (B) Scatter plots showing correlations between ACE-seq results and three replicates of EBS-seq for CRC tissue. Each dot represents a normalized average 5hmC number (EBS-seq) or a 5hmC proportion (ACE-seq) at CpG sites within sliding 100kb windows. Correlation coefficients were calculated by Pearson correlation. (C, D) Comparison of CpG positions (C) with at least one 5hmC signal and (D) with at least three 5hmC signals determined in each of three EBS-seq replicates.

**Additional file 6**: **Table S1**. Oligo sequences.

**Additional file 7**: **Table S2**. Cytosine conversion rates.

**Additional file 8**: **Table S3**. Spike-in DNA read counts.

**Additional file 9**: **Table S4**. Number of read pairs with 5hmC signals.

**Additional file 10**: **Table S5**. NGS statistics.

## Data Availability

All EBS-seq datasets, hmC-SEAL datasets, ACE-seq data of colon cancer tissue, and RNA-seq data of HEK293T cells were deposited in the Sequence Read Archive (PRJNA890016). The RNA-seq datasets used for the analysis of colorectal cancer tissues are available in the NCBI Sequence Read Archive (SRA) (http://www.ncbi.nlm.nih.gov/sra) under accession number SRR2089755.

## Abbreviations

5caC: 5-Carboxylcytosine
5fC: 5-Formylcytosine
5hmC: 5-Hydroxymethyl cytosine
5mC: 5-Methylcytosine
ACE-seq: APOBEC-coupled epigenetic sequencing
CRC: Colorectal cancer
EBS-seq: Enrichment-based single-base resolution 5hmC sequencing
hmC-SEAL: Selective 5hmC chemical labeling
hMEDIP-Seq: Antibody-based hydroxymethylated DNA immunoprecipitation
IGV: Integrative genome browser
oxBS-Seq: Oxidative bisulfite sequencing
TAB-Seq: TET-assisted bisulfite sequencing
TET: Ten-eleven translocation

## References

[1] Jin S-G, Wu X, Li AX, Pfeifer GP (2011). Genomic mapping of 5-hydroxymethylcytosine in the human brain. Nucleic Acids Res.

[2] Ficz G, Branco MR, Seisenberger S, Santos F, Krueger F, Hore TA (2011). Dynamic regulation of 5-hydroxymethylcytosine in mouse ES cells and during differentiation. Nature England.

[3] Tahiliani M, Koh KP, Shen Y, Pastor WA, Bandukwala H, Brudno Y (2009). Conversion of 5-methylcytosine to 5-hydroxymethylcytosine in mammalian DNA by MLL partner TET1. Science. Am Assoc Adv Sci.

[4] Weber AR, Krawczyk C, Robertson AB, Kuśnierczyk A, Vågbø CB, Schuermann D (2016). Biochemical reconstitution of TET1–TDG–BER-dependent active DNA demethylation reveals a highly coordinated mechanism. Nat Commun.

[5] Cimmino L, Abdel-Wahab O, Levine RL, Aifantis I (2011). TET family proteins and their role in stem cell differentiation and transformation. Cell Stem Cell.

[6] Pfeifer GP, Kadam S, Jin S-G (2013). 5-hydroxymethylcytosine and its potential roles in development and cancer. Epigen Chromatin.

[7] Stroud H, Feng S, Morey Kinney S, Pradhan S, Jacobsen SE (2011). 5-Hydroxymethylcytosine is associated with enhancers and gene bodies in human embryonic stem cells. Genome Biol.

[8] He B, Zhang C, Zhang X, Fan Y, Zeng H, Liu J (2021). Tissue-specific 5-hydroxymethylcytosine landscape of the human genome. Nat Commun.

[9] Cui X-L, Nie J, Ku J, Dougherty U, West-Szymanski DC, Collin F (2020). A human tissue map of 5-hydroxymethylcytosines exhibits tissue specificity through gene and enhancer modulation. Nat Commun.

[10] Nestor CE, Ottaviano R, Reddington J, Sproul D, Reinhardt D, Dunican D (2012). Tissue type is a major modifier of the 5-hydroxymethylcytosine content of human genes. Genome Res.

[11] Jin S-G, Jiang Y, Qiu R, Rauch TA, Wang Y, Schackert G (2011). 5-hydroxymethylcytosine is strongly depleted in human cancers but its levels do not correlate with IDH1 mutations. Cancer Res.

[12] Li W, Liu M. Distribution of 5-hydroxymethylcytosine in different human tissues. In: Veprintsev D, editor. J Nucleic Acids. SAGE-Hindawi Access to Research; 2011;2011:870726. 10.4061/2011/870726 PMC3136188 21772996

[13] Li W, Zhang X, Lu X, You L, Song Y, Luo Z (2017). 5-Hydroxymethylcytosine signatures in circulating cell-free DNA as diagnostic biomarkers for human cancers. Cell Res.

[14] Song C-X, Yin S, Ma L, Wheeler A, Chen Y, Zhang Y (2017). 5-Hydroxymethylcytosine signatures in cell-free DNA provide information about tumor types and stages. Cell Res.

[15] Booth MJ, Branco MR, Ficz G, Oxley D, Krueger F, Reik W (2012). Quantitative sequencing of 5-methylcytosine and 5-hydroxymethylcytosine at single-base resolution. Sci Am Assoc Adv Sci.

[16] Yu M, Hon GC, Szulwach KE, Song C-X, Zhang L, Kim A (2012). Base-resolution analysis of 5-hydroxymethylcytosine in the mammalian genome. Cell.

[17] Liu Y, Hu Z, Cheng J, Siejka-Zielińska P, Chen J, Inoue M (2021). Subtraction-free and bisulfite-free specific sequencing of 5-methylcytosine and its oxidized derivatives at base resolution. Nat Commun.

[18] Schutsky EK, DeNizio JE, Hu P, Liu MY, Nabel CS, Fabyanic EB (2018). Nondestructive, base-resolution sequencing of 5-hydroxymethylcytosine using a DNA deaminase. Nat Biotechnol.

[19] Xu Y, Wu F, Tan L, Kong L, Xiong L, Deng J (2011). Genome-wide regulation of 5hmC, 5mC, and gene expression by Tet1 hydroxylase in mouse embryonic stem cells. Mol Cell.

[20] Song C-X, Szulwach KE, Fu Y, Dai Q, Yi C, Li X (2011). Selective chemical labeling reveals the genome-wide distribution of 5-hydroxymethylcytosine. Nat Biotechnol.

[21] Hu L, Liu Y, Han S, Yang L, Cui X, Gao Y (2019). Jump-seq: genome-wide capture and amplification of 5-hydroxymethylcytosine sites. J Am Chem Soc US.

[22] Zeng H, He B, Xia B, Bai D, Lu X, Cai J (2018). Bisulfite-free, nanoscale analysis of 5-hydroxymethylcytosine at single base resolution. J Am Chem Soc.

[23] Li X, Shi X, Gong Y, Guo W, Liu Y, Peng C (2021). Selective chemical labeling and sequencing of 5-hydroxymethylcytosine in DNA at single-base resolution. Front Genet.

[24] Lin I-H, Chen Y-F, Hsu M-T (2017). Correlated 5-hydroxymethylcytosine (5hmC) and gene expression profiles underpin gene and organ-specific epigenetic regulation in adult mouse brain and liver. PLoS ONE.

[25] Vaisvila R, Ponnaluri VKC, Sun Z, Langhorst BW, Saleh L, Guan S (2021). Enzymatic methyl sequencing detects DNA methylation at single-base resolution from picograms of DNA. Genome Res.

[26] Robinson JT, Thorvaldsdóttir H, Winckler W, Guttman M, Lander ES, Getz G (2011). Integrative genomics viewer. Nat Biotechnol.

[27] Uribe-Lewis S, Stark R, Carroll T, Dunning MJ, Bachman M, Ito Y (2015). 5-hydroxymethylcytosine marks promoters in colon that resist DNA hypermethylation in cancer. Genome Biol.

[28] Putiri EL, Tiedemann RL, Thompson JJ, Liu C, Ho T, Choi J-H (2014). Distinct and overlapping control of 5-methylcytosine and 5-hydroxymethylcytosine by the TET proteins in human cancer cells. Genome Biol.

[29] Guler GD, Ning Y, Ku C-J, Phillips T, McCarthy E, Ellison CK (2020). Detection of early stage pancreatic cancer using 5-hydroxymethylcytosine signatures in circulating cell free DNA. Nat Commun.

[30] Chapman CG, Mariani CJ, Wu F, Meckel K, Butun F, Chuang A (2015). TET-catalyzed 5-hydroxymethylcytosine regulates gene expression in differentiating colonocytes and colon cancer. Sci Rep.

[31] Inoue A, Zhang Y (2011). Replication-dependent loss of 5-hydroxymethylcytosine in mouse preimplantation embryos. Sci Am Assoc Adv Sci.

[32] Nestor CE, Ottaviano R, Reinhardt D, Cruickshanks HA, Mjoseng HK, McPherson RC (2015). Rapid reprogramming of epigenetic and transcriptional profiles in mammalian culture systems. Genome Biol.

[33] Yin R, Mao S-Q, Zhao B, Chong Z, Yang Y, Zhao C (2013). Ascorbic acid enhances Tet-mediated 5-methylcytosine oxidation and promotes DNA demethylation in mammals. J Am Chem Soc.

[34] Li X, Liu Y, Salz T, Hansen KD, Feinberg A (2016). Whole-genome analysis of the methylome and hydroxymethylome in normal and malignant lung and liver. Genome Res.

[35] Chen K, Zhang J, Guo Z, Ma Q, Xu Z, Zhou Y (2016). Loss of 5-hydroxymethylcytosine is linked to gene body hypermethylation in kidney cancer. Cell Res.

[36] Haffner MC, Chaux A, Meeker AK, Esopi DM, Gerber J, Pellakuru LG (2011). Global 5-hydroxymethylcytosine content is significantly reduced in tissue stem/progenitor cell compartments and in human cancers. Oncotarget.

[37] Zhang L-T, Zhang L-J, Zhang J-J, Ye X-X, Xie A-M, Chen L-Y (2013). Quantification of the sixth DNA base 5-hydroxymethylcytosine in colorectal cancer tissue and C-26 cell line. Bioana Engl.

[38] Lee J-R, Kwon CH, Choi Y, Park HJ, Kim HS, Jo H-J (2016). Transcriptome analysis of paired primary colorectal carcinoma and liver metastases reveals fusion transcripts and similar gene expression profiles in primary carcinoma and liver metastases. BMC Cancer.

[39] Chen S, Zhou Y, Chen Y, Gu J (2018). fastp: an ultra-fast all-in-one FASTQ preprocessor. Bioinformatics.

[40] Kim D, Langmead B, Salzberg SL (2015). HISAT: a fast spliced aligner with low memory requirements. Nat Methods.

[41] Church DM, Schneider VA, Graves T, Auger K, Cunningham F, Bouk N (2011). Modernizing reference genome assemblies. PLoS Biol.

[42] Liao Y, Smyth GK, Shi W (2014). featureCounts: an efficient general purpose program for assigning sequence reads to genomic features. Bioinformatics.

[43] Krueger F, Andrews SR (2011). Bismark: a flexible aligner and methylation caller for Bisulfite-Seq applications. Bioinformatics.

[44] McKenna A, Hanna M, Banks E, Sivachenko A, Cibulskis K, Kernytsky A (2010). The genome analysis toolkit: a MapReduce framework for analyzing next-generation DNA sequencing data. Genome Res.

[45] Ramírez F, Dündar F, Diehl S, Grüning BA, Manke T (2014). deepTools: a flexible platform for exploring deep-sequencing data. Nucleic Acids Res.

[46] Zhang Y, Liu T, Meyer CA, Eeckhoute J, Johnson DS, Bernstein BE (2008). Model-based analysis of ChIP-Seq (MACS). Genome Biol.

[47] Heinz S, Benner C, Spann N, Bertolino E, Lin YC, Laslo P (2010). Simple combinations of lineage-determining transcription factors prime cis-regulatory elements required for macrophage and B cell identities. Mol Cell.

[48] Quinlan AR, Hall IM (2010). BEDTools: a flexible suite of utilities for comparing genomic features. Bioinformatics.

[49] Ryan D. MethylDackel [https://github.com/dpryan79/MethylDackel].

