# Supplementary figures and images for: EBS-seq: enrichment-based method for accurate analysis of 5-hydroxymethylcytosine at single-base resolution
Source: Clin Epigenetics. 2023 Mar 1;15:34. doi: 10.1186/s13148-023-01451-7 (PMC9979530; doi:10.1186/s13148-023-01451-7)

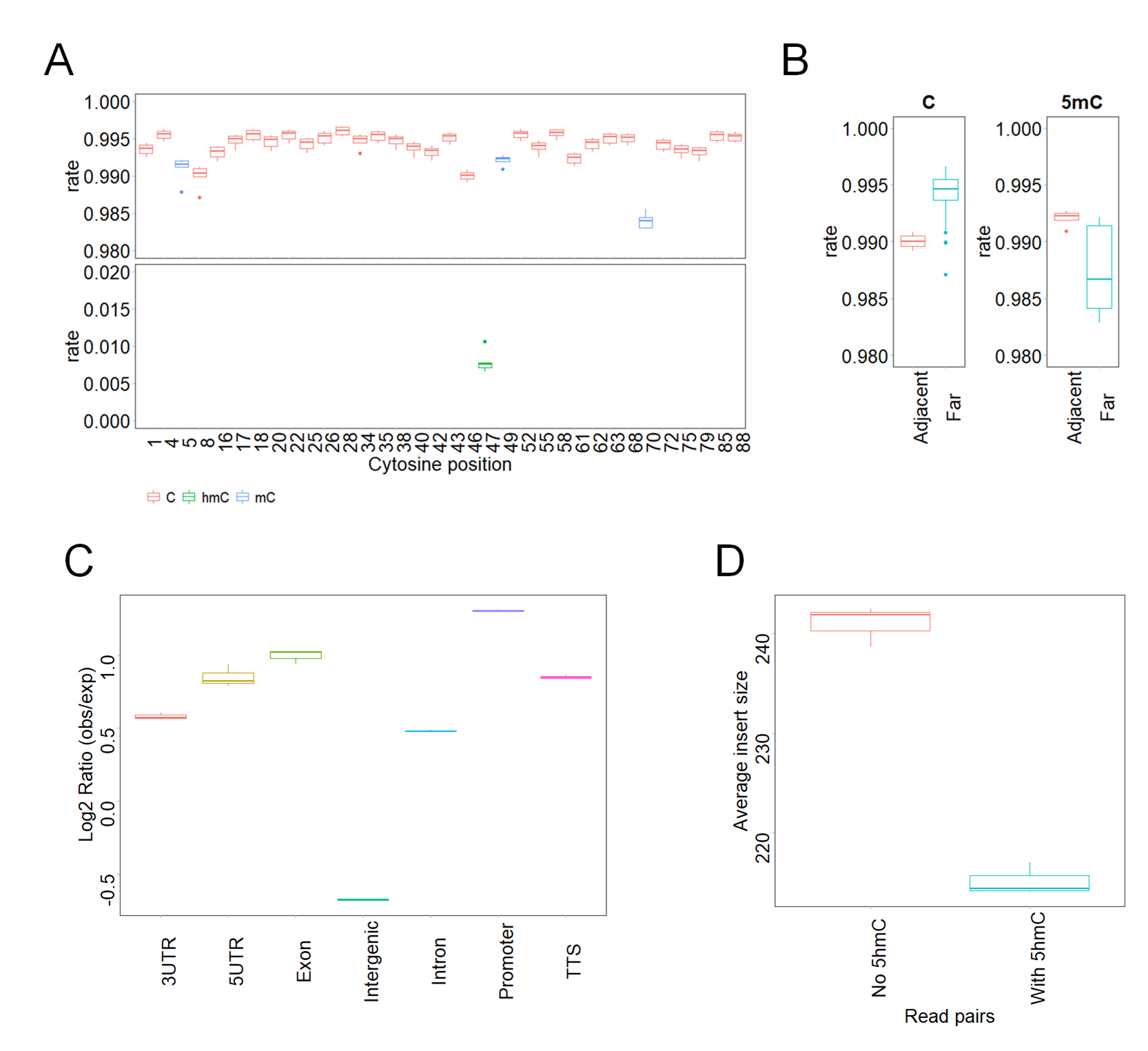

Supplement: Supplementary file 1 — Additional file 1: Fig. S1. (A) Deamination efficiency of each cytosine position in spike-in DNA. (B) Deamination efficiency of cytosines adjacent to modified 5hmC. (C) Genomic distribution of 5hmC peaks of read pairs without 5hmC. (D) Average insert size of read pairs with and without 5hmC. [file 13148_2023_1451_MOESM1_ESM.png]

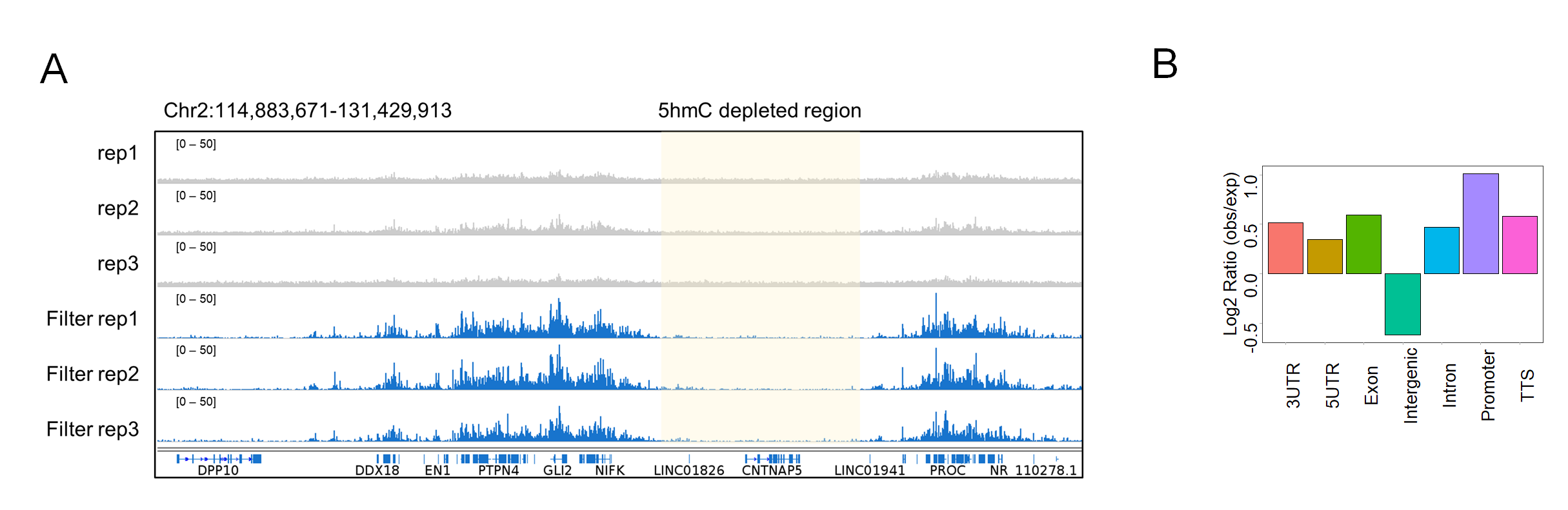

Supplement: Supplementary file 2 — Additional file 2: Fig. S2. (A) Integrative Genome Browser visualization of improved 5hmC signals of HEK293T cells in the Chr2:114,883,671–131,429,913 region. 5hmC-depleted regions are marked with yellow. Gray plots represent signals before filtering; blue plots represent signals after filtering. (B) Genomic distribution of 5hmC peaks showing enrichment in promoters and gene bodies in genomic DNA of HEK293T. [file 13148_2023_1451_MOESM2_ESM.png]

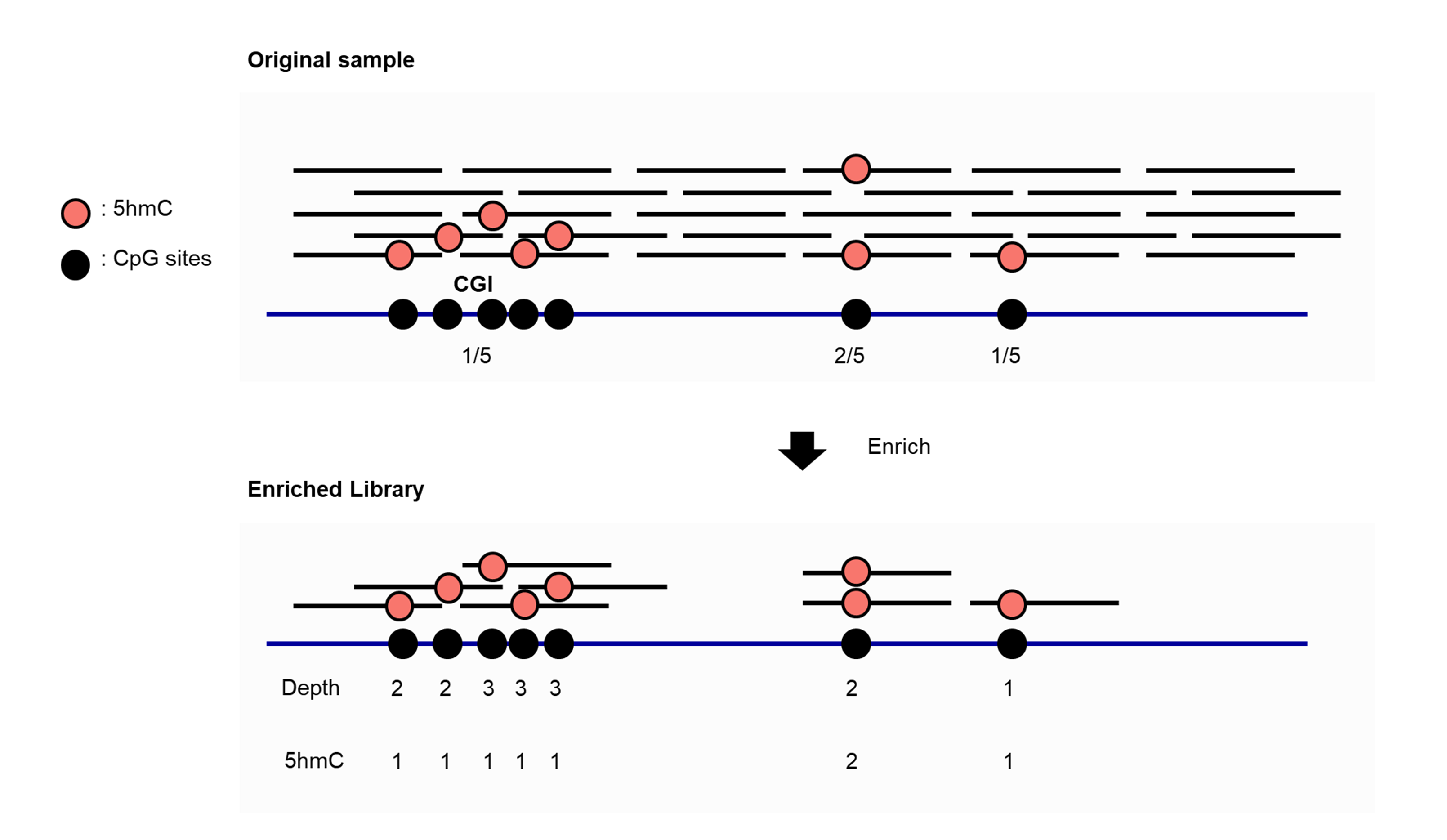

Supplement: Supplementary file 3 — Additional file 3: Fig. S3. Illustration of possible distortion caused by different regional CpG densities when using sequencing coverage to evaluate 5hmC levels. [file 13148_2023_1451_MOESM3_ESM.png]

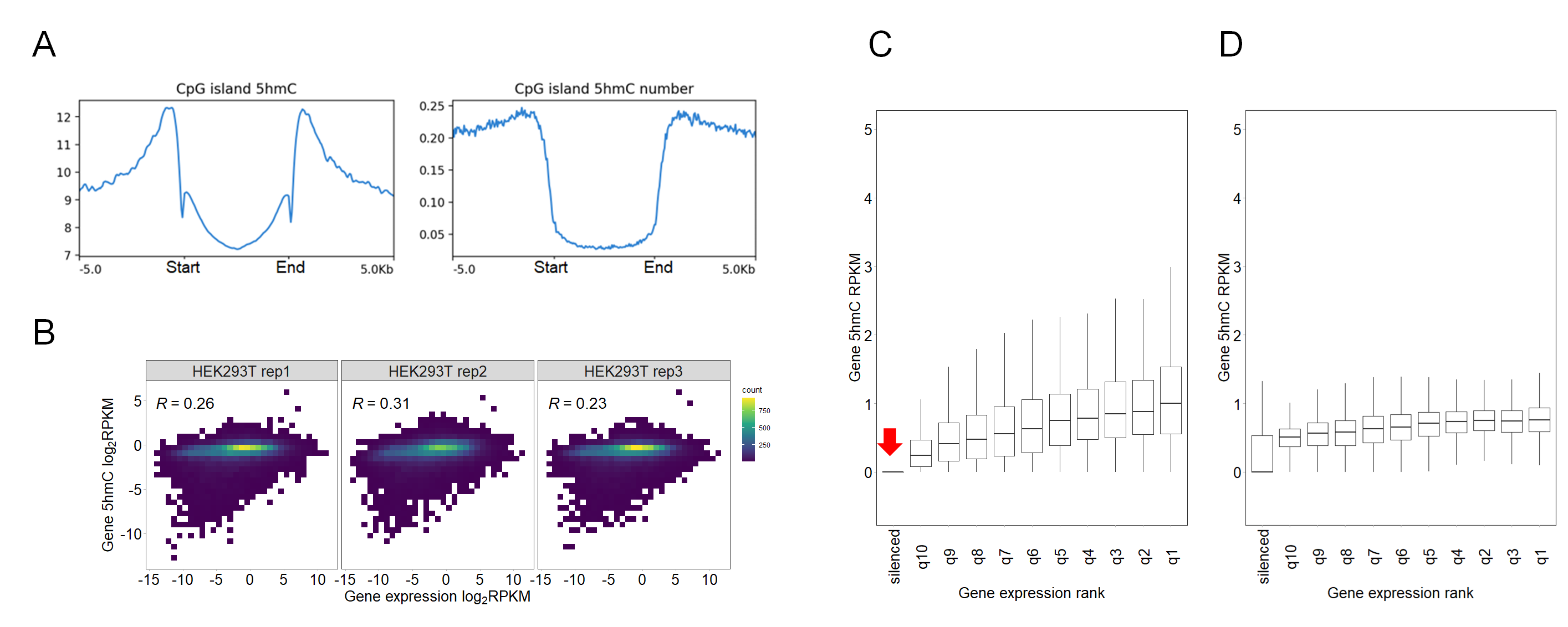

Supplement: Supplementary file 4 — Additional file 4: Fig. S4. (A) 5hmC profiles around CpG islands using normalized coverage (left) and average 5hmC numbers (right) in HEK293T cells. (B) Heatmaps showing lower correlations between gene expression and gene-body 5hmC content before noise filtering. Heatmap was created using RPKM-normalized read counts within gene bodies. Normalization was performed using the RPKM method. We observed stronger depletion of 5hmC in silenced genes (red arrow) (C) after filtering out reads without 5hmC signals than (D) before filtering. [file 13148_2023_1451_MOESM4_ESM.png]

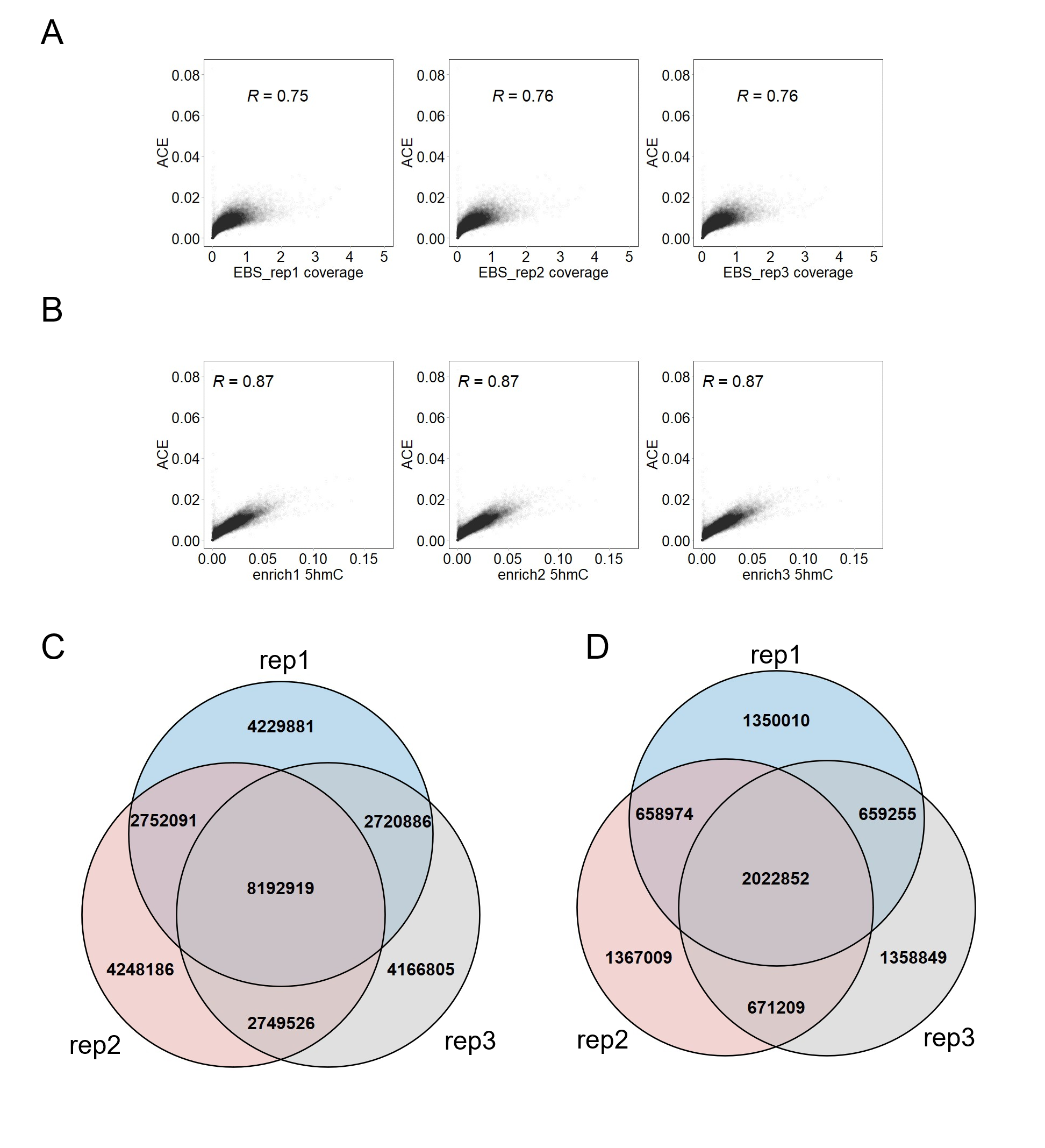

Supplement: Supplementary file 5 — Additional file 5: Fig. S5. (A) Scatter plots showing correlations between EBS-seq and ACE-seq results for CRC tissue. Each dot represents an RPKM-normalized read count (EBS-seq) or a 5hmC proportion (ACE-seq) within sliding 100kb windows. Correlation coefficients were calculated by Pearson correlation. (B) Scatter plots showing correlations between ACE-seq results and three replicates of EBS-seq for CRC tissue. Each dot represents a normalized average 5hmC number (EBS-seq) or a 5hmC proportion (ACE-seq) at CpG sites within sliding 100kb windows. Correlation coefficients were calculated by Pearson correlation. (C, D) Comparison of CpG positions (C) with at least one 5hmC signal and (D) with at least three 5hmC signals determined in each of three EBS-seq replicates. [file 13148_2023_1451_MOESM5_ESM.png]
